# Cerebrospinal fluid cortisol levels are higher in patients with delirium versus controls

**DOI:** 10.1186/1756-0500-3-33

**Published:** 2010-02-08

**Authors:** Andrew Pearson, Annick de Vries, Scott D Middleton, Fiona Gillies, Timothy O White, Ian R Armstrong, Ruth Andrew, Jonathan R Seckl, Alasdair MJ MacLullich

**Affiliations:** 1 Geriatric Medicine, University of Edinburgh, Royal Infirmary of Edinburgh, 51 Little France Crescent, Edinburgh, UK; 2 Endocrinology, University of Edinburgh, Queen's Medical Research Institute, 47 Little France Crescent, Edinburgh EH16 4TJ, UK; 3 Department of Orthopaedics and Trauma Surgery, Royal Infirmary of Edinburgh, 51 Little France Crescent, Edinburgh EH16 4SA, UK; 4 Department of Anaesthetics, Royal Infirmary of Edinburgh, 51 Little France Crescent, Edinburgh EH16 4SA, UK; 5 University of Edinburgh Centre for Cognitive Ageing and Cognitive Epidemiology, University of Edinburgh, UK

## Abstract

**Background:**

High plasma cortisol levels can cause acute cognitive and neuropsychiatric dysfunction, and have been linked with delirium. CSF cortisol levels more closely reflect brain exposure to cortisol, but there are no studies of CSF cortisol levels in delirium. In this pilot study we acquired CSF specimens at the onset of spinal anaesthesia in patients undergoing hip fracture surgery, and compared CSF and plasma cortisol levels in delirium cases versus controls.

**Findings:**

Delirium assessments were performed the evening before or on the morning of operation with a standard battery comprising cognitive tests, mental status assessments and the Confusion Assessment Method. CSF and plasma samples were obtained at the onset of the operation and cortisol levels measured. Twenty patients (15 female, 5 male) aged 62 - 93 years were studied. Seven patients were diagnosed with delirium. The mean ages of cases (81.4 (SD 7.2)) and controls (80.5 (SD 8.7)) were not significantly different (p = 0.88). The median (interquartile range) CSF cortisol levels were significantly higher in cases (63.9 (40.4-102.1) nmol/L) than controls (31.4 (21.7-43.3) nmol/L; Mann-Whitney U, p = 0.029). The median (interquartile range) of plasma cortisol was also significantly higher in cases (968.8 (886.2-1394.4) nmol/L, than controls (809.4 (544.0-986.4) nmol/L; Mann Whitney U, p = 0.036).

**Conclusions:**

These findings support an association between higher CSF cortisol levels and delirium. This extends previous findings linking higher plasma cortisol and delirium, and suggests that more definitive studies of the relationship between cortisol levels and delirium are now required.

## Background

Delirium is a severe neuropsychiatric syndrome with core features of acute onset and fluctuating course of inattention. It mainly affects older people, particularly those with chronic cognitive impairment, and is commonly precipitated by acute illness, surgery, trauma or by the effects of drugs [1]. Delirium occurs in approximately 15% of older medical inpatients and in at least one third of patients after hip fracture [2]. However, the mechanisms of delirium are under-researched and poorly understood [3].

Several lines of evidence support the hypothesis that high levels of cortisol may play a role in the pathophysiology of delirium [3,4]. Animals given high doses of glucocorticoids show cognitive deficits [5]. In experimental studies and clinical practice, high doses of cortisol or other glucocorticoids can cause inattention and other cognitive deficits [5-8]. Most patients with Cushing's disease, in which there are sustained high levels of cortisol, show neuropsychological impairments [9]. Ageing, a major risk factor for delirium, is associated with dysregulation of the hypothalamic-pituitary-adrenal (HPA) axis. This dysregulation results in higher glucocorticoid levels under basal and stressed conditions, and slower return of cortisol to baseline levels following stress [5,10,11]. Importantly, HPA axis dysregulation is pronounced in patients with dementia [12], who are at greatly increased risk of delirium [5]. Further, there is direct evidence that high plasma cortisol or HPA axis dysregulation occur in patients with delirium [3,4]. In patients aged 70-90 years undergoing abdominal surgery, those who developed delirium had substantially (>30%) higher plasma cortisol levels immediately, 24 and 48 hours after surgery [13]. O'Keeffe and Devlin [14] examined the relationship between delirium and the dexamethasone suppression test in 16 elderly patients with pneumonia. In this test, non-suppression of cortisol levels after a single dose of dexamethasone indicates dysregulation of the HPA axis. Seven of the nine patients with delirium and only one of the seven non-delirious patients were non-suppressors.

There are no published studies which have examined links between cerebrospinal fluid (CSF) cortisol levels and delirium. Determining CSF cortisol levels is potentially important because they provide a more direct and less variable measure of brain cortisol exposure than plasma levels, and because CSF cortisol levels are only variably related to plasma levels [15]. CNS cortisol levels are modified by multiple factors including the degree of blood brain permeability [16], the activity of the multidrug-resistance gene type-1 P-glycoprotein [17], and steroid metabolising enzymes within the brain, such as 11β-hydroxysteroid dehydrogenase type 1 [18].

More generally, there are very few studies which have examined CSF in delirium [19-21], despite the obvious value of such studies. This may be partly because that obtaining CSF samples in such patients presents considerable ethical and practical challenges. One method which avoids many of these challenges is to obtain CSF samples in patients with hip fracture, who have a high prevalence of delirium, at the time of induction of spinal anaesthesia for fracture repair. CSF collection at the time of spinal anaesthesia has been used in one study on hip fracture patients [22], which found higher CSF IL-8 levels in hip fracture patients versus elective arthroplasty controls, but has not yet been used in the study of delirium.

In the present study we tested the hypothesis that delirium is associated with higher CSF and plasma cortisol levels in older patients with acute hip fracture.

## Methods

### Participants

We studied patients with acute hip fracture over 60 years old who were awaiting surgery under spinal anaesthesia. Patients gave written, informed consent. Exclusion criteria were residence in an institution, a documented history of dementia or depressive illness, and corticosteroid therapy within the last 10 weeks. The study was approved by the Scotland A Research Ethics Committee.

### Delirium Assessment

Delirium assessments were performed pre-operatively, no more than 16 hours before the operation, and before any pre-operative sedative medication. Each assessment comprised a standard battery of scales (Mini-Mental State Examination, digit span, the Delirium Symptom Interview, the Memorial Delirium Assessment Scale and the Confusion Assessment Method) validated for the diagnosis of delirium [23]. In this battery these combined assessments are used to complete the standard and widely-used Confusion Assessment Method (CAM), an established instrument for the detection of delirium [24]. The CAM comprises a four-point algorithm for delirium diagnosis. For a positive diagnosis patients have to show (a) an acute onset or fluctuating course, and (b) inattention, and they must also demonstrate (c) disorganised thinking and/or (d) altered arousal. The comprehensive, validated battery we used allows each of these features to be defined, through a combination of patient assessments (interview, clinical observation, and cognitive testing), and information from staff, carers, and casenotes.

### Sample Collection and Assays

Samples of CSF (2 ml) and clotted blood (10 ml) were taken before administration of the spinal anaesthestic agent. Samples were spun at 1000 g for 10 minutes at 4°C. To check for blood contamination in the CSF, the pellet was resuspended in 100 ul phosphate buffer saline followed by a cytospin. All samples were stored at -80°C before analysis. Plasma cortisol assays were performed with a Cortisol-^125^I RIA (ImmunChem™, MP Biomedicals), with a detection limit of 22.4 nmol/l. CSF cortisol levels were determined with a cortisol ELISA kit (Salimetrics, USA). The detection range for this kit is 0.08 - 82.8 nmol/L.

### Statistical analysis

Group differences in cortisol levels were analysed with the Mann-Whitney U test (two-tailed). Non-parametric correlation (Spearman's rho) was used to examine the relationship between CSF and plasma cortisol levels.

## Results

Twenty-seven patients were recruited. Two did not undergo spinal anaesthesia after recruitment, and an additional patient had their operation postponed. Four further patients were excluded because of blood contamination of the CSF specimens. The age range of the 20 included subjects in the study was 62-93 years (mean 80.6, SD 8.0). As expected in this group of older hospital inpatients, many had comorbid disease (Table 1). Operations started between the hours of 0800 and 1500. The mean duration between sample acquisition and centrifugation was 1.6 hours (SD 1.1).

**Table 1:** Subject characteristics, and cerebrospinal fluid and plasma cortisol levels

	**ID no**.	Age	Sex	Major comorbidities	CSF cortisol (nmol/L)	Plasma cortisol (nmol/L)
CASES	1	76	F	-	54.9	886.2

	2	83	F	HT, COPD	63.9	968.8

	3	82	F	IHD, HT	18.8	669.4

	4	85	F	IHD, HT	245.0	3053.7

	5	93	F	IHD, type 2 DM	67.1	1394.4

	6	70	M	IHD, type 2 DM	40.4	910.6

	7	81	F	HT, COPD	102.1	1104.2

						

CONTROLS	8	81	F	IHD, HT	29.2	698.3

	9	79	M	IHD, COPD	46.4	857.4

	10	93	F	IHD	40.2	838.3

	11	90	F	-	37.8	897.1

	13	87	F	-	79.9	1097.0

	14	79	M	-	31.1	746.0

	15	62	F	rheumatoid arthritis	21.0	1075.6

	16	77	F	HT	33.3	463.4

	17	88	M	-	19.5	481.0

	18	88	F	IHD	46.5	809.4

	19	71	F	-	22.5	497.7

	20	73	F	HT	16.1	1100.3

	22	79	M	IHD	27.0	590.3

**Figure 1:**
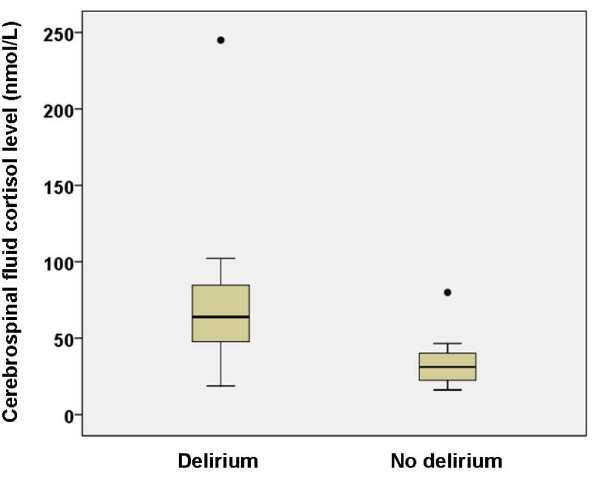
**Cerebrospinal fluid cortisol levels in patients with and without delirium**.

Seven patients had delirium according to the Confusion Assessment Method. The mean ages of cases (81.4 (SD 7.2)) and controls (80.5 (SD 8.7)) were not significantly different (p = 0.88). Median (interquartile range) CSF cortisol levels were significantly higher in subjects with delirium (63.9 (40.4-102.1) nmol/L) than those without (31.4 (21.7-43.3) nmol/L; Mann-Whitney U, p = 0.029). (Figure 1). The median (interquartile range) of plasma cortisol levels was also significantly higher in cases (968.8 (886.2-1394.4) nmol/L) than in controls (809.4 (544.0-986.4) nmol/L; Mann Whitney U, p = 0.036).

Plasma and CSF cortisol levels correlated at rho = 0.59 (p = 0.006). The ratios of CSF to plasma cortisol were not statistically different between the groups (Mann Whitney U, p = 0.122).

## Discussion

The novel finding in this study was that CSF cortisol levels were significantly higher in hip fracture patients with delirium versus controls. Plasma cortisol levels were also significantly higher in cases versus controls. The direction of causation cannot be determined in an observational study, but these findings are consistent with the hypothesis that high brain cortisol levels are associated with delirium.

Higher levels of cortisol occurring after hip fracture are an essential part of the systemic response to injury. Consistent with this, the CSF cortisol levels in the present study were generally considerably higher than those in healthy younger control subjects (mean 12.3, SEM 1.26)[15], in healthy older people (mean 8.3, SEM 0.55)[25], or in patients with Alzheimer's disease (mean = 21.90, SD = 8.1)[26]; the levels were around half of those in patients with septic meningitis (median 133 nmol/L (interquartile range 59-278)[27]. Two patients with delirium had extremely high CSF cortisol levels (cases 4 and 7), of 102.1 and 245.0 nmol/L. The causes of this degree of elevation is uncertain, but very high plasma cortisol levels of several thousand nmol/L are commonly observed in critically ill patients [28]. These extreme levels may reflect the severity of illness in combination with loss of feedback regulation.

There is good evidence that the degree of elevation of cortisol, even within the 'stress' range, is important in causing cognitive dysfunction. For example, in healthy young males, high doses of administered cortisol over four days (reaching plasma levels of 700-800 nmol/L) caused verbal memory impairment [8] and in another study continuous infusion of high-dose cortisol caused impairments in working memory [6]. Thus, although elevated levels of cortisol are a critical component of the physiological response to injury, pathologically high cortisol levels might be more likely to impair cognitive functioning, and even precipitate delirium in vulnerable patients [13]. Another important factor may be individual differences in vulnerability to cortisol-induced cognitive dysfunction [3]. Indeed, in this study levels of cortisol in cases and controls showed some overlap.

Some limitations of the study should be noted. The sample size was relatively small. Dementia is a risk factor for delirium and is also associated with higher CSF cortisol levels[26], and so is a potential confounder, though in the present study patients with known dementia were excluded. However, future studies would benefit from more detailed screening of patients, and retrospective informant history, for example with the Informant Questionnaire on Cognitive Decline in the Elderly (IQCODE)[29]. CSF sampling occurred at different times of the day; however this may not be important because of the loss of diurnal rhythm of cortisol in the early phase after a fracture [30]. Another limitation is that some delirium assessments took place the evening before surgery, several hours before the cortisol measurements. Future studies should aim to conduct these assessments and measurements as close together as possible. Additionally, possible interactions between drugs known to precipitate delirium, and cortisol levels, should be explored.

These results suggest that patients with delirium have higher levels of cortisol in the CSF and plasma. Should a causal role for cortisol be established, this would suggest a potential role for antiglucocorticoid therapies in delirium.

## Abbreviations

HT: hypertension; COPD: chronic obstructive pulmonary disease; IHD: ischaemic heart disease; type 2 DM: type 2 diabetes mellitus.

## Competing interests

The authors declare that they have no competing interests.

## Authors' contributions

AM conceived the study and led its design and coordination. AP, SM, FG and AM carried out the delirium assessments, assays, and contributed to statistical analyses and writing of the manuscript. AdV, TW, IA, RA and JS participated in the design and coordination of the study and contributed to writing the manuscript. All authors read and approved the final manuscript.
